# Editorial: Employees' resistance and turnover intentions to the use of digital technologies: from the perspective of leadership

**DOI:** 10.3389/fpsyg.2023.1222889

**Published:** 2023-08-03

**Authors:** Abdul Waheed Siyal, Chen Hongzhuan, Imran Khan, Gang Chen

**Affiliations:** ^1^College of Economics and Management, Nanjing University of Aeronautics and Astronautics, Nanjing, China; ^2^Department of Management Sciences, The Islamia University of Bahawalpur, Bahawalpur, Pakistan; ^3^Department of Data Science and Management Engineering, School of Management, Zhejiang University, Hangzhou, China

**Keywords:** digital technologies, employees resistance, technology complexity, turnover intentions, leadership style

Digital technologies have become deeply integrated into our daily lives through smartphones and other computing devices. Individuals carry out all personal, professional, and social matters through the use of digital technologies. As a routine, individuals use these means for organizational communications such as regular report submission, customer relationship management, complaint resolution, and feedback, etc. The proliferation of digital technologies has reshaped the conduct of behavioral research by shifting the traditional research setting into the digital realm. Unsurprisingly, digital transformation exposes organizations to numerous ethical, administrative, and leadership issues that adversely affect organizational green development.

While considering the challenges of digital technologies that might impact employees' well-being and health and trigger resistance toward new technologies, leaders could effectively manage employees by allowing them to raise their voice and by putting relative issues on the table and praising employees for well-performed jobs. The level of employees' resistance to novel technologies could be substantially affected by leadership styles. For example, unrealistic planning by leaders would obviously make it hard for employees to take on new technologies and achieve assigned targets by the given deadlines, thus resulting in low performance. This poor performance could demotivate employees and trigger resistance to new technologies, leading them to exit their jobs.

The current Research Topic helped to study complex human behaviors to better understand how technological resistance impairs employees' psychological well-being and performance at work. The published results portray the mechanisms leaders could employ to minimize technological complexity and cope with employees' resistance against constant technological changes, which ultimately result in turnover intentions.

## Author contributions

All the co-topic editors have played their relevant roles to successfully complete this Research Topic. All authors contributed to the article and approved the submitted version.

